# Design and Characterization of a 52K SNP Chip for Goats

**DOI:** 10.1371/journal.pone.0086227

**Published:** 2014-01-22

**Authors:** Gwenola Tosser-Klopp, Philippe Bardou, Olivier Bouchez, Cédric Cabau, Richard Crooijmans, Yang Dong, Cécile Donnadieu-Tonon, André Eggen, Henri C. M. Heuven, Saadiah Jamli, Abdullah Johari Jiken, Christophe Klopp, Cynthia T. Lawley, John McEwan, Patrice Martin, Carole R. Moreno, Philippe Mulsant, Ibouniyamine Nabihoudine, Eric Pailhoux, Isabelle Palhière, Rachel Rupp, Julien Sarry, Brian L. Sayre, Aurélie Tircazes, Jun Wang, Wen Wang, Wenguang Zhang, and the International Goat Genome Consortium

**Affiliations:** 1 INRA, UMR444, Laboratoire de Génétique Cellulaire, Castanet-Tolosan, France; 2 ENVT, UMR444, Laboratoire de Génétique Cellulaire, Castanet-Tolosan, France; 3 INRA, Sigenae, Castanet-Tolosan, France; 4 INRA, GeT-PlaGe, Genotoul, Castanet-Tolosan, France; 5 Wageningen University, Animal Breeding and Genomics Centre, Wageningen, The Netherlands; 6 Kunming Institute of Zoology, Chinese Academy of Sciences, State Key Laboratory of Genetic Resources and Evolution, Kunming, China; 7 Illumina Inc., Hayward, California, United States of America; 8 Utrecht University, Faculty of Veterinary Medicine, Utrecht, The Netherlands; 9 Malaysian Agricultural Research and Development Institute, Strategic Livestock Research Centre, Kuala Lumpur, Malaysia; 10 INRA, UR 0875, Mathématiques et Informatique Appliquées Toulouse, Castanet-Tolosan, France; 11 AgResearch, Invermay Agricultural Center, Mosgiel, New Zealand; 12 INRA, UMR1313 Génétique Animale et Biologie Intégrative, Jouy en Josas, France; 13 AgroParisTech, UMR 1313 Génétique Animale et Biologie Intégrative, Jouy en Josas, France; 14 INRA, UPR0631, Station d’Amélioration Génétique des Animaux, Castanet-Tolosan, France; 15 INRA, UMR1198 Biologie du Développement et Reproduction, Jouy en Josas, France; 16 ENVA, UMR1198 Biologie du Développement et Reproduction, Jouy en Josas, France; 17 Virginia State University, Department of Biology, Petersburg, Virginia, United States of America; 18 Bejing Genome Institute, BGI-Shenzhen, Shenzhen, China; 19 Inner Mongolia Agricultural University, Inner Mongolia Key Laboratory of Animal Genetics, Breeding and Reproduction, Hohhot, Inner Mongolia, China; Auburn University, United States of America

## Abstract

The success of Genome Wide Association Studies in the discovery of sequence variation linked to complex traits in humans has increased interest in high throughput SNP genotyping assays in livestock species. Primary goals are QTL detection and genomic selection. The purpose here was design of a 50–60,000 SNP chip for goats. The success of a moderate density SNP assay depends on reliable bioinformatic SNP detection procedures, the technological success rate of the SNP design, even spacing of SNPs on the genome and selection of Minor Allele Frequencies (MAF) suitable to use in diverse breeds. Through the federation of three SNP discovery projects consolidated as the International Goat Genome Consortium, we have identified approximately twelve million high quality SNP variants in the goat genome stored in a database together with their biological and technical characteristics. These SNPs were identified within and between six breeds (meat, milk and mixed): Alpine, Boer, Creole, Katjang, Saanen and Savanna, comprising a total of 97 animals. Whole genome and Reduced Representation Library sequences were aligned on >10 kb scaffolds of the *de novo* goat genome assembly. The 60,000 selected SNPs, evenly spaced on the goat genome, were submitted for oligo manufacturing (Illumina, Inc) and published in dbSNP along with flanking sequences and map position on goat assemblies (i.e. scaffolds and pseudo-chromosomes), sheep genome V2 and cattle UMD3.1 assembly. Ten breeds were then used to validate the SNP content and 52,295 loci could be successfully genotyped and used to generate a final cluster file. The combined strategy of using mainly whole genome Next Generation Sequencing and mapping on a contig genome assembly, complemented with Illumina design tools proved to be efficient in producing this GoatSNP50 chip. Advances in use of molecular markers are expected to accelerate goat genomic studies in coming years.

## Introduction

Goat was one of the first domesticated animals, around 10,500 years ago in the Fertile Crescent (for a review, see [1]). According to the FAO, the world goat population has been estimated to be around 921 million animals, with an increase of more than 20% during the last ten years. Goats are a source of milk, meat and fibre and are adapted to a wide range of grazing environments. To date, however, they lack genomic research tools available in cattle and sheep.

Goat genome knowledge has benefited from different sources: the development of generic resources such as a BAC large inserts genomic library [2], mapping of microsatellites and genes [3], local resources developed in order to make progress in a specific genome region (for example, researches on Polled Intersex Syndrome have allowed progress on the chromosome 1 genetic map [4], [5]) and comparative cytogenetic analysis between goat genome and related species as reviewed in [6]. Fontanesi and co-authors have also used bovine tiling arrays to characterize copy number variations in goats [7].

Recently, the development of next generation sequencing (NGS) allowed *de novo* sequencing of the goat genome, which in turn offered an opportunity to create the International Goat Genome Consortium (IGGC, www.goatgenome.org) in 2010, whose aims were to consolidate research efforts at the international level. The Goat Genome reference sequence has been published [8] and is available to the scientific community through a web interface and mirror (http://goat.kiz.ac.cn/GGD/). A RH panel has also been developed [9]. Alongside these two new tools to speed up development of genetic and physical animal maps, single nucleotide polymorphism (SNP) panels allow screening the genetic variability of a species and thus open the way towards their use for genomic selection [10]. SNP chips have already been developed for several domestic animal species [11] and cattle are the best funded with a clear use for genomic selection. Tools for cattle include low (3K, 7K [12]), moderate (50K [13]) and High density SNP chips (628K & 777K). 50–60K SNP chips have also been developed for sheep, pigs [14] and chickens [15] and a 700–800K chip is under development for sheep (James Kijas, personal communication). One of the challenges of developing a SNP chip is ensuring utility of the chip in a wide variety of breeds. In 2010, goat SNP detection projects were underway in: France, Netherlands and Malaysia/Canada, all prioritizing native breeds. IGGC proposed the consolidation of these SNP detection projects towards the goal of creating an international SNP chip. The objective was to design a moderate density chip (50–60K) consisting of markers evenly spaced across the goat genome and segregating with high to medium allele frequencies in each of six breeds (Alpine, Saanen, Creole, Boer, Katjang and Savanna).

## Results

### Goat Reference Genome

**Figure 1:**
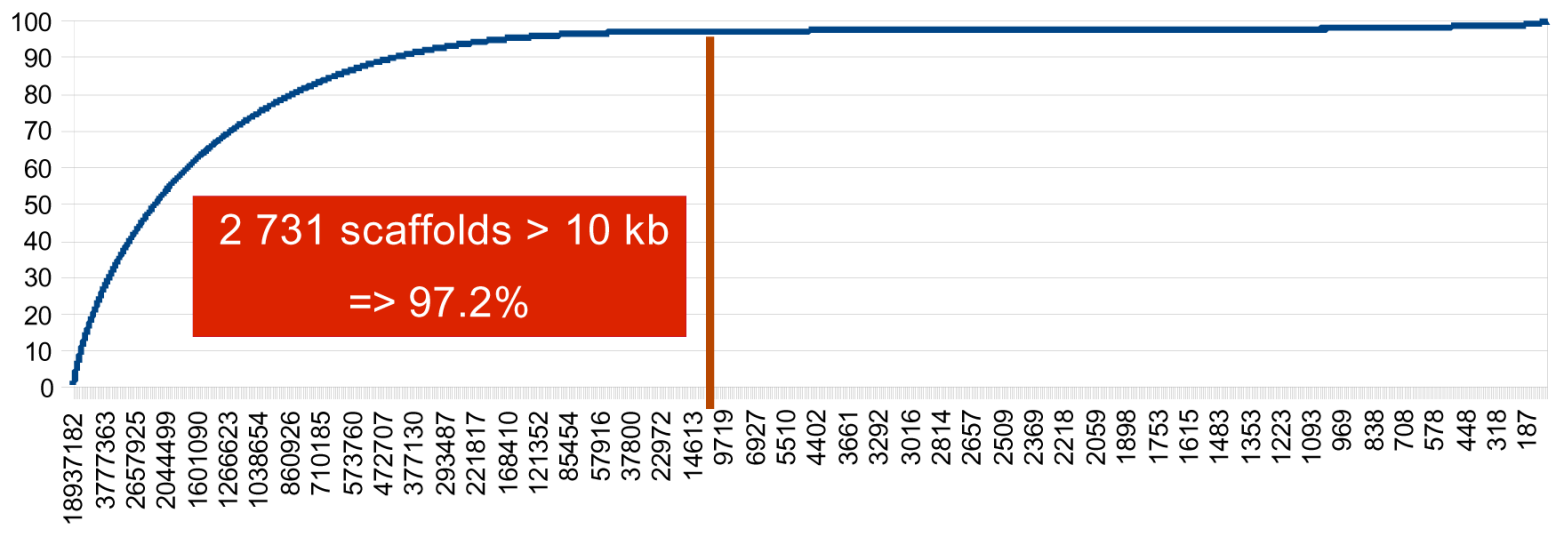
Goat Genome scaffolds assembly. The goat genome scaffolds were sorted by decreasing size (x-axis) and the cumulative proportion of the assembled genome was plotted on the y-axis for all the scaffolds. The vertical line shows that >10kb scaffolds represent 97.2% of the assembled goat genome.

Scaffolds of the *de novo* assembled goat genome were made available by BGI sequencing center (www.genomics.cn) to allow mapping of the flanking sequences of the identified SNPs. The assembly consisted of 285,379 scaffolds representing 2,665,022,236 bp. Since a Yunling female goat was sequenced to create the assembly, no representation of the Y chromosome was included in the sequence. As shown in Figure 1, there were 2,731 scaffolds larger than 10 kb, representing 97% of the total assembly. These were used for mapping sequences generated for SNP discovery, to ensure complete genome coverage.

### SNP Discovery

SNP discovery projects were underway in three laboratories, before they were consolidated within the IGGC. This explains why the SNP discovery was achieved through two different pipelines, for milk and mixed breeds (Alpine, Saanen and Creole) in one project and meat breeds (Boer, Katjang and Savanna) in another project.

For dairy and mixed breeds, sixteen animals (seven Alpine, three Creole and six Saanen) were sequenced using a HiSeq2000 (Illumina, Inc) and produced 80 to 220 million high quality (passing filter) 100 bp reads per animal. This resulted in a sequencing coverage of 13.5 to 26X depending on the breed, after filtration of the BAM files. Another set of 17 Dutch Saanen animals was used to construct a RRL library. Sequencing from this RRL library resulted in 120 million 76bp sequences. The sequence coverage, using those data, after BAM filtration, was 2.85X on average in the genomic regions that were covered with one read at least.

SNP discovery was performed for each breed separately after which the results were combined. A minimum of two reads per allele was mandatory in the definition of a SNP not including the reference, with a minimum sequencing depth of six and a maximum depth of 100, to avoid repeated regions. These criteria have also been used with success in cattle [13], while in chicken and pigs [14], [15], a minimum of three reads were used for SNP validation. The number of discovery SNPs for each of the breeds is reported in Table 1 and ranged from 2.1 million to 6.3 million. Insertions/deletions (INDEL) (200,000 to 620,000 depending on the breed) were also detected (data not shown).

**Table 1:** SNP identified in the five breeds or breed pool and in ESTs.

	Alpine	Boer	Creole	Katjang/Savanna	Saanen	ESTs
**Alpine**	**6 271 599**	701 503	832 959	774 144	3 154 048	3 133
**Boer**		**2 276 196**	184 853	1 321 410	594 634	1 092
**Creole**			**2 123 300**	200 083	794 951	644
**Katjang/Savanna**				**2 641 668**	662 060	1 163
**Saanen**					**5 466 080**	2 714
**ESTs**						**6 929**

The number on the diagonal is the number of SNPs found in a breed (Alpine, Boer, Creole, Saanen), breed pool (Katjang/Savanna) or in ESTs. Off diagonals are the number of SNPs shared between the two respective breeds.

For meat breeds, sequencing was performed on two pools of animals (20 Boer goats in pool 1 and 20 Savanna together with 24 Katjang goats in pool 2). Over 680 million sequences (106 Gbp) were obtained for each pool, resulting in an average coverage of 35X. After alignment to sheep and cattle reference genomes, SNP discovery was performed within each pool and resulted in 4,001,890 SNPs (among which 1,073,183 SNPs were specific to pool 1 and 1,476,768 SNPs specific to pool 2). A minimum of three reads per allele was used to define a SNP, not including the reference. Around 90% of the four million SNPs and their flanking sequences were successfully aligned with goat sequence scaffolds. The number of these discovery SNPs is reported in Table 1 for each breed together with the number of SNPs that are common to two breeds.

Additional SNPs were identified in expression data, from published ESTs and RNASeq projects (see acknowledgements section) and included in the candidates for final SNP selection. The detection procedure described in the methods section was followed by mapping on the goat scaffolds and resulted in a 7,008 SNP list.

Regarding PRNP, casein and DGAT1 genes, already known as important for milk composition and disease resistance, regions orthologous to cattle were identified on goat scaffolds 881, 980 and 2304 respectively (Table S1) and extended to ensure encompassing the gene regulating regions. The “PRNP region” was set as the interval (1,640,000–1,663,000) on scaffold881, “casein regions” as the intervals (293,900–329,750; 390,000–410,500; 510,000–525,000) on scaffold980 and “DGAT1 region” as interval (45,000–71,000) on scaffold2304. Within those regions, all Infinium II (see definition below) SNPs were retained. Already published SNPs in PRNP, casein and DGAT1 were aligned on goat scaffolds and all but three were found in the previous selection (Table S2). Finally, 21 SNPs were retained in PRNP region, 56 SNPs in casein regions and seven SNPs in DGAT1 region.

A total of 11,924,638 variants were tracked including 1,229,120 Indels and 10,695,518 discovery SNPs. For each variant, Illumina Assay Design Tool (ADT) score, Infinium type (I or II, see below) and allele frequency was stored and used for final SNP selection.

### SNP Selection and Chip Design

Illumina iSelect 60K designs can include both Infinium II SNPs (A/C; A/G; T/C; T/G), that require a single bead type, and Infinium I SNPs (A/T; C/G) that require two bead types for genotyping [16]. We opted to select only Infinium II SNPs, to maximise the number of genotyped polymorphisms, within the allocated space on the SNP chip.

**Figure 2:**
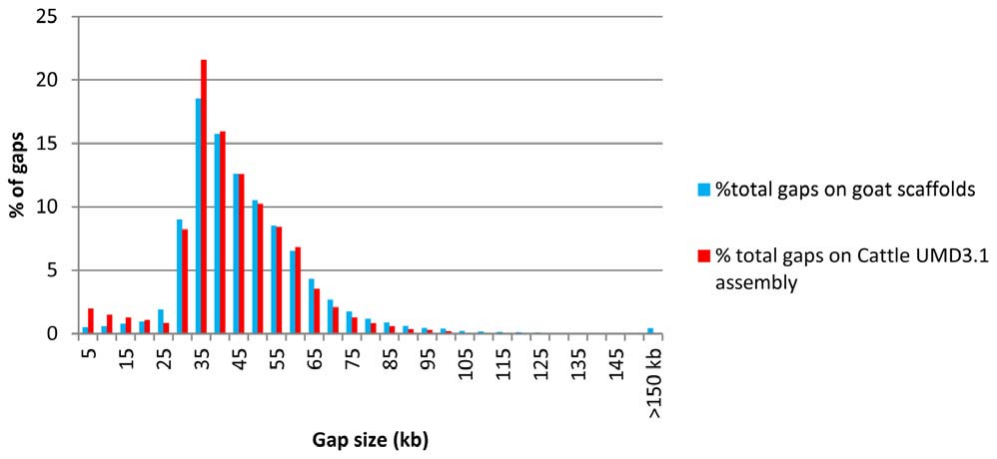
SNP spacing on the goat scaffolds. Spacing between the selected SNPs was calculated and the percentage of gaps (total number of gaps is 59,030 on goat scaffolds and 62,693 on UMD3.1 cattle assembly) is shown (y-axis) in each 5kb class ranging from 5 to 150kb (x-axis).

**Figure 3:**
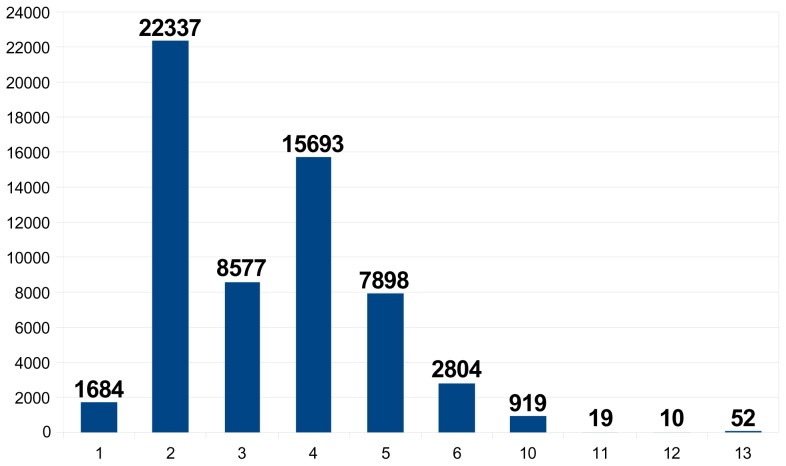
SNPs by category in final design. The number of selected SNPs is indicated for each of the following categories. 1: SNP detected in an EST. 2: two alleles detected in the five considered breeds. 3: two alleles detected in Alpine and Saanen and Creole and (Boer or Savanna). 4: two alleles detected in two of the three milk and mixed breeds (Alpine, Saanen, Creole) and in Boer and Savanna. 5: two alleles detected in Alpine and Saanen and Creole. 6: two alleles detected in three out of the five breeds. 10: two alleles detected in each of the two milk breeds (Saanen and Alpine). 11: two alleles detected in one milk breed (Saanen or Alpine) and one meat breed (Creole or Boer or Katjang/Savanna). 12: two alleles detected in at least two meat breeds (Creole and Boer or Katjang/Savanna). 13: two alleles detected in one milk breed (Saanen or Alpine).

Since the average spacing of markers on a 50–60K SNP chip is about 60,000 bp, we disregarded SNPs mapping on contigs of fewer than 10,000 bp. To maximise the success rate of the beadpool, we also eliminated INDELs or SNPs that a) represented >2 alleles b) were within 20bp from another variant c) had an ADT score <0.8 or d) had an estimated MAF <0.2 in breeds of interest. This list of 537,145 SNP was ranked using increasing category number (see material and methods) and decreasing MAF. Class 1 SNPs were detected in ESTs and all included. The following ten classes corresponded to a decreasing number of breeds in which the SNP is observed. The first selection of 60,000 SNPs was done with spacing along the genome using an in-house algorithm (available on request). More than 97% of the SNPs were heterozygous in at least three breeds. To capture some remaining large genomic regions without SNPs, 1,000 SNPs of this first list were replaced by an alternative SNP, presenting either a higher number category or a <0.2 MAF or an <0.8 ADT score. Finally, as shown in Figure 2, there were only 26 scaffolds regions larger than 150kb without a SNP. As described in Figure 3, the 60,000 selected SNPs consisted in 1,684 SNPs identified in ESTs, 22,337 (38%) SNPs heterozygous in all the breeds, 24,270 (40.5%) SNPs heterozygous in four different breeds, 10,702 (18%) SNPs heterozygous in three different breeds and 1,000 other SNPs. Thus, 96% of the SNPS were heterozygous in at least three breeds and also heterozygous in at least one milk breed (Alpine or Saanen) and one meat or mixed breed (Creole, Katjang, Savanna or Boer).

**Figure 4:**
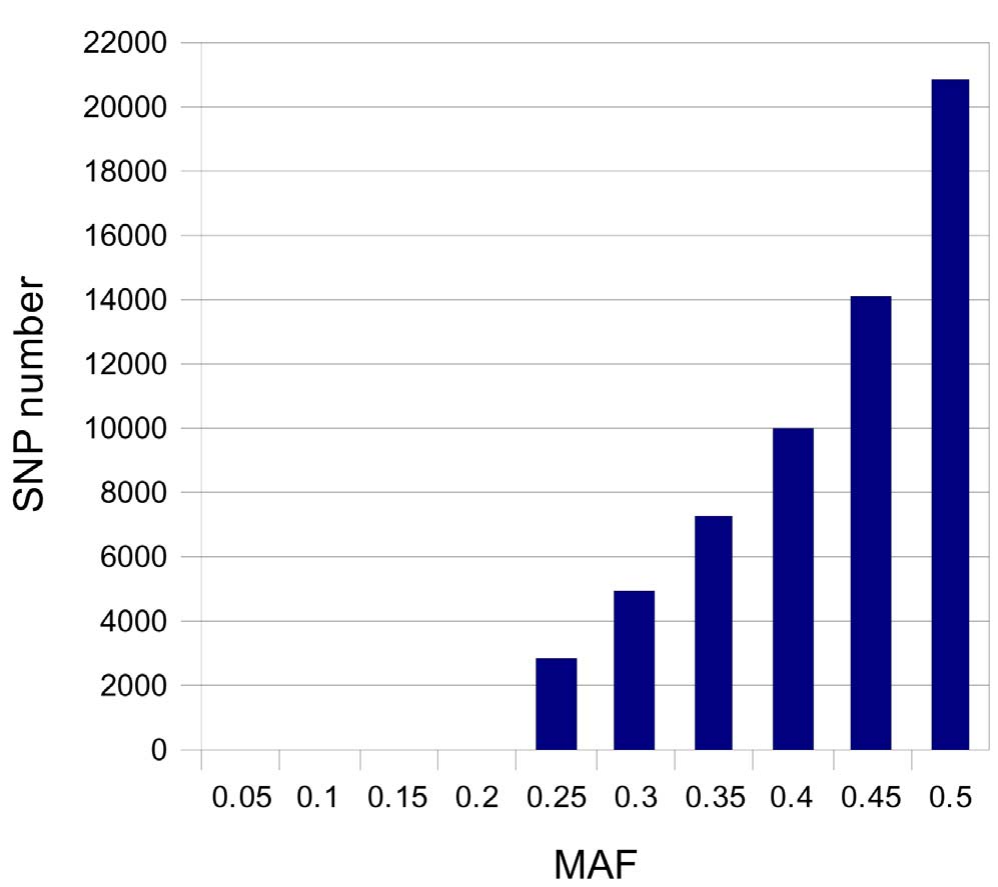
Distribution of estimated MAFs of the selected SNPs. The MAF for all the 60,000 selected SNPs was estimated based on the read counts for the two alleles.

Estimated MAFs of the selected SNP ranged from 0.05 to 0.5 with 50% of the selected SNP with a >0.420 MAF, as shown in Figure 4.

To check the spacing procedure on a closely related assembled genome, the selected SNP flanking sequences were aligned on cattle UMD3.1 assembly. A total of 59,001 SNP flanking sequences were aligned on the cattle genome. As shown in Figure 2, the selected SNPs were evenly spaced on the cattle genome, which shows high co-linearity with the goat genome. The distribution of the intervals between selected SNPs, either on goat scaffolds or on the cattle genome showed that 92% of the SNPs were at a [30kb-90kb] distant from the next adjacent SNP.

For the 60,000 selected SNPs, the variants were submitted to dbSNP under the handle IGGC with batch ID “CAPRI-BATCH1” (http://www.ncbi.nlm.nih.gov/projects/SNP/snp_viewBatch.cgi?sbid=1057128 and File S1). The submission also included 60bp flanking sequences on each side of the variant site, estimated MAF value, Illumina_score, Illumina_state (successful or not) and position on goat scaffolds, CHIR_1.0, Sheep_OARV2 and Bovine_UMD3.1. Unique dbSNP Submitter SNP (ss) accession numbers were assigned for each variant, including those on the goatSNP50 chip, and they are listed in File S2. The corresponding Reference SNP (rs) numbers were assigned for dbSNP Build 136 release in December, 2012. As the goat reference genome evolves, it is possible a small fraction of rs numbers may be updated in future dbSNP release if evidence suggests a different assignment.

The file containing this information is also downloadable from the IGGC website (www.goatgenome.org).

### Performance of the SNP Chip

From the 60,000 selected SNP sequences, Illumina successfully synthesized 53,347 SNPs (89%). This beadpoool was tested with 288 animals. 281 animals were successfully genotyped on 52,295 loci (87.15% of the submitted SNPs and 98% of the synthesized SNPs) that had a minimum average call rate of 99.83% (Table 2). These genotypes were used to construct the cluster file (downloadable from the IGGC website and also available from Illumina) that can be used to assign genotypes from the raw data files of genotyping experiments. Thirty two SNPs of the 1,052 poor performing loci have been annotated, as they display multiple clusters that could have biological meaning. Five hundred and fourty three SNPs of the 52,295 final SNPs have also been manually annotated in the cluster file. Five hundred and twenty four loci have a pattern that suggest the presence of either a nearby polymorphism or a deletion and 19 display multiple clusters, that suggest possible copy number polymorphism.

**Table 2:** Average call rate and >5%MAF SNPs for the cluster file breeds.

Breed	Samples	SNPs MAF>/ = 0.05	Av call rate
**Alpine**	53	51339	0.9990
**Angora**	26	47195	0.9986
**Boer**	30	48494	0.9989
**Creole**	38	50216	0.9988
**Jinlan**	13	45648	0.9983
**Katjang**	13	33873	0.9987
**Saanen**	57	51689	0.9989
**Savanna**	20	46629	0.9990
**Skopelos**	27	50908	0.9987
**Yunling**	1	17335	0.9995
**Total**	281		0.9988

For each breed used for the chip validation and cluster file definition, the number of samples, the number of >5%MAF SNPs and the average call rate are indicated.

The results of MAF >5% are indicated in Table 2 for the ten breeds of these 281 animals. Even if results are not well-estimated for breeds with lower than 20 samples, it should be noted that Angora and Skopelos that were not used for SNP discovery show segregation for a >47,000 set of SNPs with >5% MAF.

## Discussion

The GoatSNP50 chip described in this paper has been validated as a useful tool for a variety of goat breeds, with >78% SNPs segregating in seven breeds, including Angora and Skopelos, that had not been used for SNP discovery. This chip has already been successfully used for genetic diversity studies on Boer, Cashmere and Rangelang goats [17]. This utility across non-discovery breeds is the consequence of using six goat breeds for SNP discovery with different characteristics (including milk, meat and mixed breeds), origins, and grazing environments. Our procedures to identify SNPs and estimate their MAF proved to be reliable, with a >98% successful loci, comparable to the rate observed in chicken (95%, [15]) and somewhat higher to that observed in cattle (88%, [13]) or pigs (89%, [14]). However, the estimates of SNPs declared as polymorphic by the various authors are quite difficult to compare since: the number of samples genotyped and proportion of genotyped samples used for SNP discovery, differed. Factors contributing to the high conversion rate include: a high sequencing depth (13.5 to 35X, depending on the breed) and the selection of high MAF candidate SNPs across breeds. This high sequencing coverage on the whole genome, when compared to Sanger sequencing strategies previously used, has been obtained through NGS technologies. This sequencing coverage, combined with the choice of high MAF SNP, had already proven its efficiency, on reduced representations of the genome [15].

Technology failures during probe synthesis resulted in 11% loss of SNPs that were submitted to design resulting in 89% conversion of the pre-manufacturing content which was slightly below expected conversion of working assays based upon the weighted average ADT design score of.95 leading to the expectation that 95% of the assays would convert to working assays. The reason for the failure of a subset of the probes to appear in the final post-manufacturing content is independent of the likelihood of a sequence to convert to a working design due to the random nature of the assembly of beads into wells and the minimum redundancy threshold in the Infinium manufacturing process for each probe. If a probe was manufactured successfully, but the number of beads representing that probe were not represented in a minimum redundancy of over 5x (for an average of 15x redundancy of all probes), then it was removed *via* the quality checking process by Illumina. This results in a random failure that is unrelated to the sequence and design of the probes themselves.

In addition, because we did not have goat genome input into the ADT tool, we were not able to do a check on probe design based upon expected repetitive, low diversity or otherwise known problematic regions of the genome. As a result, any scores that come from the design as “species: Other” will be slightly inflated compared to whether we had the genome check integrated into the design process.

50–60K SNP chips are primarily used for finding association between markers and phenotypes. To achieve this well, both a uniform distribution of the SNPs on the genome and the complete coverage of the genome are required. A large excess of SNPs is required to be able to evenly space the SNPs on the genome. Groenen and co-authors [15] proposed “a rule of thumb” that this number be 10-fold higher than the targeted number of the final chip. With about 10 million SNPs in our database, we were easily able to achieve a uniform spacing. This source of high-quality SNPs will also be useful for further studies and tool development: e.g. we will have a large number of SNPs available for fine-mapping in subsequent studies. However, the sequencing depth we have achieved is not sufficient to identify the majority SNPs with low MAF. These are a source of SNPs which are valuable for association studies as emphasized by Gorlov et al. [18]. With the use of whole genome sequence at high coverage, complete coverage of the genome was achieved using contigs >10kb and a preliminary assembly of the goat genome. This highlights the efficiency of using draft genome assemblies for SNP chip designs. Only 26 regions larger than 150kb remained without a designed SNP on the goat scaffolds. To independently check this spacing, we successfully mapped 59,000 SNPs out of the 60,000 selected SNPs on the cattle genome, supporting the already described co-linearity of cattle and goat genomes. On the cattle genome, 260 regions larger than 150kb without a SNP were observed. This can be explained by i) differences between cattle and goat genomes, ii) the use of >10kb goat scaffolds only that represent 97% of the goat genome, 3% of the genome remaining unassembled in our sequences.

### Conclusions

The combined strategy of using mainly whole genome NGS and mapping on a contig genome assembly, coupled with Illumina designing tools proved to be efficient when creating the GoatSNP50 chip. We hope that with this tool goat genomics studies can now advance rapidly.

## Materials and Methods

### Ethics Statement

DNA samples from this study came from three Institutes in France, Netherlands and Malaysia. Neither sperm collection nor blood sampling was performed specifically for this study. Sperm collection was performed on bucks by Capgenes, which obtained the authorization from DGAL (Direction Générale de l’ALimentation) FR CC 860. Sperm collection was made by Artificial Insemination stations, and we used extra doses from this collection. Blood samples were taken from commercial farms. Animals did not belong to any experimental design but were sampled by veterinarians and/or under Veterinarian supervision for routine veterinary care; extra samples were requested when blood sampling occurred.

### Animals and DNA Samples

High quality genomic DNA was extracted from sperm of seven Alpine, three Creole and six French Saanen bucks. Briefly, the content of three straws from the same animal was pelleted (10,000g, 5 minutes) after dilution in 500 µL of 10mM TrisHCl, 10mM EDTA, 150mM NaCl pH = 7.5 buffer and rinsed in the same buffer. Disulphide bonds of protamines were reduced with DTT and then proteins digested through the addition of 200 µL of 10mM Tris-HCl (pH 7.5), EDTA 10mM, NaCl 100mM, SDS, 2%, 0.1M DTT, incubation 1.5h at 65°C and then addition of 20 µL of 10mg/mL proteinase K and further incubation at 37°C overnight with agitation. The next day, two extractions with an equal volume of Tris-HCl (pH = 8) saturated phenol, followed by two chloroform extractions were performed and the final supernatant was precipitated with sodium acetate and ethanol. After centrifugation (10,000 g, 30 min), the DNA pellet was dissolved in 500 µL of Tris-HCl 10mM, EDTA 0.1mM, pH = 7.4. DNA was extracted out of sperm from 17 Dutch Saanen bucks using a similar protocol. Genomic DNA was extracted from 300 µL of blood samples of 20 Boer goats, 20 Savanna goats and 24 Katjang goats individually using Promega Wizard Genomic DNA Purification System. DNA quantity and purity were measured using the Nanodrop ND1000. Possible degradation was inspected on an agarose gel and only high quality undegraded DNA samples were used to prepare the DNA pools.

### Construction of Reduced Representation Libraries

For detection of SNPs on Dutch Saanen bucks, restricted representation libraries (RRL) were constructed as described by Kraus et al [19]. In short, equal amounts of DNA from the 17 bucks were combined into two pools each consisting of 25 µg of DNA. Aliquots of 5 µg for each pool were digested with either *Alu*I or *Hha*I (10 units per reaction, Pharmacia). The digested pools in Orange loading dye (Fermentas) were size-fractionated on precast 10% polyacrylamide in 1xTBE with the Criterion™ Cell (BioRad). After staining, the target fragment size range of 110–130 bp was sliced out of the gel. The gel slice was sheared by nesting a 0.5ml eppendorf tube (with a hole in the bottom formed with a needle) containing the gel slice inside a 2ml eppendorf tube, and centrifuged at 14000 rpm for 2 minutes. The sheared gel pieces were covered with 300ul DNA recovery buffer (8mM Tris pH 8.0, 0.08mM EDTA, 1.25M ammonium acetate), vortexed, and eluted at 4°C overnight, followed by 15 minutes incubation at 65°C. The DNA was recovered using the Montage DNA gel extraction devices (Millipore) and resuspended in DNA hydration solution (Gentra Systems).

### Next Generation Sequencing

Each of the 16 DNAs from Alpine, Creole or Saanen goat was sequenced independently on an Illumina HiSeq2000 machine using one lane for each animal. 3µg of DNA was used for each animal to construct libraries following manufacturer’s protocol (Illumina TruSeq DNA sample prep). Then, libraries were quantified and sequenced on one lane for each on an Illumina HiSeq2000 sequencer using standard Illumina protocol (TruSeq SBS kits v3), with 100 bp paired-end reads and ∼250 bp insert length.

Two genomic DNA pools were made for meat goats: pool 1 from 20 Boer goats and pool 2 from 20 Savanna goats and 24 Katjang goats. Each DNA sample was quantified three times (using Hoechst dye and fluorometer) and equal amount of genomic DNA from each sample based on the average concentration of the three readings were used to make DNA pools. The pools were whole genome shotgun sequenced on an Illumina Genome Analyzer IIx machine using the standard Illumina protocol, with 78 bp paired-end reads and ∼300 bp insert length.

The genomic RRLs of the 17 Dutch Saanen bucks (*Alu*I and *Hha*I) were combined and prepared using the Illumina Sample Preparation kit [20] and sequenced with the Illumina GAII, Illumina Inc., USA with 76 bp paired end reads.

Access to high-throughput sequences can be requested from research teams and subjected to signing of a Data Transfer Agreement.

### SNP Discovery and Characterization

*De novo* sequencing of the goat genome [8] lead to primary assembly into goat scaffolds and further assembly into 30 pseudo-chromosomes (CHIR_1.0). These two datasets are downloadable from www.goatgenome.org website. CHIR_1.0 is also downloadable from http://goat.kiz.ac.cn/GGD/. Alpine, Saanen and Creole FASTQ sequences were aligned on the goat scaffolds with **BWA** software [21] (“aln” algorithm with default settings). The resulting SAM format files were processed using **samtools view**, **sort** and **merge** functions [22]. Alignments with a unique location and a >30 mapping quality were kept and.bcf files were generated using **samtools mpileup** and **bcftools** view tools with the following parameters: minimum depth = 6, maximum depth = 100 (to avoid repeated regions of the genome), minimum number of reads for each allele = 2. SNP MAFs were estimated by counting the number of reads of each allele for the whole dataset (from the 16 animals).

Data from the two pools of meat goats were aligned on cattle UMD3.1 and sheep Oarv2∶0 reference genomes, available respectively on http://www.cbcb.umd.edu/research/bos_taurus_assembly.shtml and http://www.livestockgenomics.csiro.au/sheep/websites, using **MosaikAlign** [**22**] with default values except mode (-m) unique, using parameters as mismatch (-mm) 12, alignment quality threshold (-mhp) 100, alignment candidate threshold (-act) 35. SNP discovery within and across pools in the genomic regions well aligned with the reference genomes was done using **Mosaik** [23], **samtools** (**samtools** to generate pileup file and **samtools.pl varFilter** with default values to filter SNPs), and DNA LandMarks (http://www.dnalandmarks.ca/) in-house algorithm. The quality filtering for SNP discovery were a SNP quality of at least 20 and minimum three reads of each allele. Approximately 300 bp of consensus flanking sequence of aligned goat reads were extracted together with the variant and the MAF was calculated as above. These sequences were aligned on the goat scaffolds using **BWA** (“bwasw” algorithm with default settings).

SNPs were also detected in RNASeq projects (see acknowledgements section) conducted either on gonads (GENIDOV programme) or mammary gland (LGS programme). Sequencing data generated by 454/Roche platform were *de novo* assembled and several stringent filters were applied to resulting contigs to predict SNPs, as described in [24]. The SNPs and their flanking sequences were then mapped on the goat scaffolds.

Finally, previously published SNP in genes of interest (caseins, PRP, DGAT) were added. Briefly, the genomic sequence of cattle CSN1S1, CSN2, CSN1S2, CSN3, PRP and DGAT1 genes were retrieved from ensemble genome browser (http://www.ensembl.org/, release 64) and were blasted [25] using default parameters on the goat scaffolds and the blast results were filtered with the following parameters: hits>1000pb and % identity>90% to determine the orthologous scaffold part (Table S1). Within these enlarged regions, to get sure to encompass regulating regions of the gene, all the SNP were selected. Additional SNP probes (if different from the previous ones) were designed using already described variants in the following publications: [26], [27], [28] for PRNP gene and [29] for casein genes.

For all the detected SNPs, 120 bp of flanking sequences were extracted. Using **Illumina Assay Design Tool**, the type of the SNP (Infinium I or II) and the technical quality of the SNP for use on Illumina BeadChips (scored [0,1], using “Other” genome as a reference) were assessed.

Categories were defined to characterize the variant:

1: SNP detected in an EST.
2: two alleles detected in the five considered breeds.
3: two alleles detected in Alpine and Saanen and Creole and (Boer or Katjang/Savanna).
4: two alleles detected in two of the three milk and mixed breeds (Alpine, Saanen, Creole) and in Boer and Katjang/Savanna.
5: two alleles detected in Alpine and Saanen and Creole.
6: two alleles detected in three out of the five breeds.
10: two alleles detected in each of the two milk breeds (Saanen and Alpine).
11: two alleles detected in one milk breed (Saanen or Alpine) and one meat breed (Creole or Boer or Katjang/Savanna).
12: two alleles detected in at least two meat breeds (Creole and Boer or Katjang/Savanna).
13: two alleles detected in one milk breed (Saanen or Alpine).
20: other SNPs.
90: INDELs.

### Construction of a SNP Database

For all the detected variants, the following characteristics of the detected SNP were stored in an in-house database:

- Goat scaffold name
- Goat scaffold position
- Base in the goat reference genome and in all the analyzed breeds
- 2 by 60 bp of flanking sequence
- Estimated MAF (Minimum Allele Frequency): approximated here by counting the number of reads for each allele
- Illumina SNP score: as determined by **Illumina Assay Design Tool** (ADT), using the closest to goat available genome as a reference: cattle; ranging from 0 to 1 and evaluating the probability of success of the SNP assay
- Illumina SNP type: I (A/T or G/C) requiring two two probes or II (other SNPs) requiring only one probe
- Distance to the previous and the next SNP (bp)

### Selection of the Final SNP List

The following criteria were used to select a first list of SNPs:

scaffold >10,000 bp, biallelic, minimum 20bp each side without another variant, MAF >0.2, *Illumina score >0.8*, Infinium II SNP. This list was ranked using increasing category and decreasing MAF.

An in-house algorithm was used to select a SNP list maximizing both the rank criteria presented before and the genome coverage. The algorithm uses the rank sorted SNPs and the scaffold length array as inputs. During the process it fills an array storing starts and end positions of already covered genome locations and populates the list of selected SNPs. The algorithm checks a SNP at each cycle. For each SNP, it first checks if it is located in an already covered area. In this case, it processes the next SNP. If the SNP is located in an uncovered area, the flanking area (28,500 bp on each side or scaffold end position if reached) is added to the covered positions array and the SNP is added to selected SNP list. Several flanking area sizes were tested. The one which was chosen: 28,500 produced a list of close to 59,000 SNPs.

The remaining 1,000 SNP were manually chosen to cover large genomic regions which did not include highly ranked SNPs.

SNP flanking sequences were aligned on cattle UMD3.1 assembly using **BWA** (“**bwasw”** algorithm with default settings).

The final 60,000 selected SNP list was submitted to Illumina.

### Quality Control of the Chip

To assess the quality of the SNP chip, a set of 288 DNAs (285 animals that included four parent-parent-child trios and three duplicates) were used. It included most of the animals that had been used for SNP discovery and the animal used for reference genome sequence. Additional animals were added to represent genetic diversity of international goat breeds. This breed set included 53 Alpine, 26 Angora, 30 Boer, 38 Creole, 16 Jinlan, 15 Katjang, 59 Saanen, 20 Savanna, 27 Skopelos and 1 Yunling goat, with four trios. Genotyping of these 288 DNAs was used to assess the performance of the successfully synthesized SNP assays and generate cluster files.

## Supporting Information

Table S1 **PRNP, casein and DGAT1 orthologous regions on cattle and goat genomes.** For each gene, chromosome number and location are given on cattle UMD3.1 assembly and scaffold number and location are given on goat scaffolds.
(XLSX)

Table S2 **SNPs in PRNP, casein and DGAT1 genes.** For each SNP, exon number, flanking sequence and variant, scaffold name, position on the scaffold and ss number in dbSNP are indicated.
(XLSX)

File S1 **60,000 SNP chip design. This “CAPRI-BATCH1” file contains information on the 60,000 selected SNPs as it was submitted to dbSNP (** http://www.ncbi.nlm.nih.gov/projects/SNP/snp_viewBatch.cgi?sbid=1057128 **).** The submission includes 60bp flanking sequences on each side of the variant site, MAF value, Illumina_score, Illumina_state (successful or not) and position of the variant on goat scaffolds (Chinese_assembly_v1 column) and goat CHIR_1.0 assembly, (Chinese_assembly_v2_pseudo column), Sheep_OARV2 and Bovine_UMD3.1.
(GZ) Click here for additional data file.

File S2 **60,000 SNP ss names.** This file contains the correspondence between SNP name in File S1 and NCBI_ss# name for the 60,000 selected SNPs.
(GZ) Click here for additional data file.
