# Correction: Design and Characterization of a 52K SNP Chip for Goats

**DOI:** 10.1371/journal.pone.0152632

**Published:** 2016-03-24

**Authors:** Gwenola Tosser-Klopp, Philippe Bardou, Olivier Bouchez, Cédric Cabau, Richard Crooijmans, Yang Dong, Cécile Donnadieu-Tonon, André Eggen, Henri C. M. Heuven, Saadiah Jamli, Abdullah Johari Jiken, Christophe Klopp, Cynthia T. Lawley, John McEwan, Patrice Martin, Carole R. Moreno, Philippe Mulsant, Ibouniyamine Nabihoudine, Eric Pailhoux, Isabelle Palhière, Rachel Rupp, Julien Sarry, Brian L. Sayre, Aurélie Tircazes, Jun Wang, Wen Wang, Wenguang Zhang, International Goat Genome Consortium

We have forgotten to thank our collaborators who provided the Creole samples. The Acknowledgements section should read:
